# Comparative Effectiveness of Management Strategies for Stage IV Prostate Cancer: A Narrative Review

**DOI:** 10.7759/cureus.89396

**Published:** 2025-08-05

**Authors:** Okelue E Okobi, Tobechukwu J Okobi, Francis Okobi, Rita Kimble, Cherish D Adesola, Ebere M Nwachukwu, Michael O Oluwalana, Kingsley O Ozojide, Stanley Ezulike

**Affiliations:** 1 Family Medicine, Larkin Community Hospital Palm Springs Campus, Miami, USA; 2 Family Medicine, IMG Research Academy and Consulting LLC, Homestead, USA; 3 Internal Medicine, BronxCare Health System, New York, USA; 4 Research and Development, Covance, Hanover, USA; 5 Research and Development, University of Maryland, Laurel, USA; 6 Pharmacy, D'youville School of Pharmacy, Beaumont, USA; 7 Kinesiology-Exercise Science, Georgia Southern University, Statesboro, USA; 8 Medicine, Memorial University of Newfoundland, St. John's, CAN; 9 Public Health, Nottingham Trent University, Nottingham, GBR; 10 Internal Medicine, Chukwuemeka Odumegwu Ojukwu University Teaching Hospital, Awka, NGA

**Keywords:** abiraterone, adt, cabazitaxel, docetaxel, enzalutamide, metastatic prostate cancer, personalized treatment, radiotherapy, stage iv prostate cancer

## Abstract

Stage IV prostate cancer (PCa) refers to a disease that has metastasized beyond the prostate gland to distant sites, such as bones, visceral organs, or non-regional lymph nodes. While early attempts at curative therapy were occasionally made in oligometastatic cases, current guidelines uniformly recommend palliative-intent management once true metastatic spread is confirmed. Over the past decade, treatment paradigms have shifted from androgen deprivation therapy (ADT) monotherapy to earlier intensification with combination regimens including chemo-hormonal therapy and next-generation hormonal agents to improve survival and quality of life (QoL). This narrative review evaluates the comparative effectiveness of ADT, chemotherapy, novel hormonal agents, and palliative radiotherapy in patients with stage IV prostate cancer. A structured literature search of PubMed, Embase, and the Cochrane Library was conducted for English-language studies published between January 2015 and May 2025. Search terms included “stage IV prostate cancer,” “metastatic prostate cancer,” “androgen deprivation therapy,” “docetaxel,” “enzalutamide,” “abiraterone,” “palliative radiotherapy,” and “systemic therapy.” Eligible studies involved adult male patients with stage IV prostate cancer receiving systemic therapies or radiotherapy; preclinical studies, case reports, and non-metastatic populations were excluded.

ADT remains the foundational therapy for metastatic disease. In hormone-sensitive, high-risk patients, combination therapy with agents such as enzalutamide or abiraterone has been shown to significantly improve progression-free survival (PFS) and overall survival (OS). Chemotherapy, particularly docetaxel, provides survival benefits in patients with high-volume metastatic hormone-sensitive disease, as demonstrated in landmark trials. Cabazitaxel, typically used in the castration-resistant setting, offers favorable tolerability compared to docetaxel rechallenge. Among novel hormonal agents, enzalutamide is often preferred in older or comorbid patients due to superior cost-effectiveness and a more favorable metabolic and cardiovascular profile compared to abiraterone. Palliative radiotherapy contributes to symptom control and local disease management, especially when guided by biomarkers such as circulating tumor cells (CTCs). However, variability in study design, patient characteristics, and prior treatments complicates direct comparisons. Managing stage IV prostate cancer requires an individualized, palliative-focused strategy. This review highlights the value of early combination approaches based on disease volume, patient performance status, comorbidities, and personal preferences. ADT remains the therapeutic cornerstone, with systemic intensification and selective radiotherapy offering additive benefits in appropriately chosen patients.

## Introduction and background

Prostate cancer (PCa) is among the most prevalent cancers in men in the world, with an increasing rate in the older generation [1]. Although early‑stage disease often offers curative interventions, such as radical prostatectomy (RP) or localized radiotherapy, a substantial proportion of patients progress to stage IV disease. According to the 2025 European Association of Urology (EAU) classification, stage IV prostate cancer includes any tumor with documented distant metastases to bones, visceral organs, or non-regional lymph nodes [2]. This advanced stage is further subclassified into oligometastatic prostate cancer, defined by a limited number (≤5) of metastatic lesions, and high-volume metastatic disease, characterized by visceral metastases or ≥4 bone lesions with at least one beyond the axial skeleton. High-volume metastatic disease is further stratified into hormone-sensitive prostate cancer (HSPC), which initially responds to androgen deprivation therapy (ADT), and castration-resistant prostate cancer (CRPC), which progresses despite castrate levels of testosterone. Progressing beyond this point, the disease is often viewed as incurable, and attempts to treat it shift focus from curative intent to controlling symptoms, slowing disease progression, and maintaining quality of life (QoL) [2].

Deciding on the most appropriate treatment strategy for stage IV prostate cancer remains a complex and clinically significant challenge. Although multiple therapeutic options are now available, including androgen deprivation therapy (ADT), chemotherapy (docetaxel or cabazitaxel), novel hormonal agents (enzalutamide or abiraterone), palliative radiotherapy for symptomatic metastases, and supportive or palliative care services, there is ongoing uncertainty about how best to sequence or combine these modalities, particularly in specific patient subgroups such as those with hormone-sensitive versus castration-resistant disease or high-volume versus low-volume metastases [3]. Each strategy carries distinct implications for survival, toxicity, and quality of life, necessitating individualized decisions guided by disease characteristics, comorbidities, and evolving clinical evidence. Given these complexities and the rapid evolution of treatment standards over the past decade, a comprehensive review is timely and necessary to synthesize current evidence and inform clinical decision-making [4].

The management of stage IV prostate cancer requires strategic, patient-centered consideration of how treatment modalities impact quality of life, mental health, and functional outcomes [1,2]. Novel hormonal agents, such as enzalutamide and abiraterone, have demonstrated superior survival benefits; however, their use may be limited in certain populations due to associated risks, including cardiovascular complications and generalized fatigue [3-5]. ADT remains a mainstay for disease control but poses significant challenges in elderly or frail patients, particularly those with comorbidities, due to adverse effects such as cognitive impairment, osteoporosis, and cardiovascular risk [1]. Chemotherapeutic agents, including cabazitaxel and docetaxel, can be effective in patients with a high metastatic burden and good functional status, yet their toxicity profiles often impair tolerability and negatively affect quality of life [6-10].

Regardless of the chosen modality, patients with stage IV prostate cancer frequently experience substantial treatment-related side effects and a significant symptom burden [5]. Evidence supports the early integration of palliative care to mitigate these effects, particularly in addressing ADT-related complications such as depression, fatigue, and musculoskeletal decline in older adults [5,6]. As such, the timely incorporation of palliative strategies during active treatment is essential to maintaining functional status and quality of life in this population [7].

Despite the growing availability of systemic therapies and evolving guidelines, considerable uncertainty remains regarding optimal treatment sequencing, combination strategies, and personalization of care across subgroups (e.g., hormone-sensitive versus castration-resistant and low- versus high-volume disease) [11,12]. Prior reviews have often focused on isolated treatment modalities, excluded real-world data, and lacked integration of palliative care considerations, limiting their relevance to routine clinical practice [13,14]. This review aims to address these gaps by providing a comprehensive synthesis of both clinical trial and real-world evidence, encompassing survival, tolerability, and the evolving role of palliative radiotherapy and supportive care to inform individualized, evidence-aligned management strategies in stage IV prostate cancer.

In the absence of randomized controlled studies, ADT at the early-stage initiation fails to provide a major survival benefit compared to deferred ADT therapy [5]. Since more therapeutic options are becoming available in management strategies and the current clinical guidelines are changing, such a narrative review seeks to address and compare the main management strategy effectiveness. The research question is as follows: "in adult male patients with stage IV prostate cancer (P), how do androgen deprivation therapy, chemotherapy, novel hormonal agents, palliative radiotherapy, best supportive care, and palliative care (I/C) compare in terms of overall survival (OS), quality of life, and symptom control (O)?" This review aims to support clinicians and patients in making informed, evidence-based decisions by synthesizing current research on personalized approaches to the management of advanced disease. Through an integrated analysis of existing literature, the review seeks to guide the selection of tailored treatment strategies that align with individual patient profiles and clinical contexts.

## Review

Methods

To ensure transparency and reproducibility, this narrative review was conducted using a structured literature search strategy. We searched three electronic databases, PubMed, Embase, and the Cochrane Library, for English-language studies published between January 2015 and May 2025. The search utilized the following key terms and Boolean combinations: “stage IV prostate cancer,” “metastatic prostate cancer,” “androgen deprivation therapy,” “docetaxel,” “cabazitaxel,” “enzalutamide,” “abiraterone,” “palliative radiotherapy,” “supportive care,” and “palliative care.”

Studies were included if they involved adult male patients aged 18 years or older with a clearly defined diagnosis of stage IV (metastatic) prostate cancer. Eligible publications comprised clinical trials, cohort studies, cost-effectiveness analyses, and real-world evidence that evaluated one or more key interventions. These interventions included ADT, chemotherapy, next-generation hormonal agents, palliative radiotherapy, and palliative or supportive care. Studies were required to report on at least one clinically relevant outcome, such as overall survival (OS), progression-free survival (PFS), symptom control, or treatment-related adverse events.

We excluded case reports, preclinical studies, and narrative or systematic reviews that lacked original data. Additionally, studies involving non-metastatic populations or those with unclear disease staging were not considered. Non-English language publications were also excluded to maintain consistency and ensure accessibility for the review team.

Two independent reviewers screened titles and abstracts for relevance, conducted full-text reviews, and extracted data using a standardized template. Data fields included study design, patient demographics, treatment modalities, key clinical outcomes (OS, PFS, QoL, and symptom relief), and adverse events. Disagreements were resolved by consensus. A narrative review approach was chosen over a systematic review due to the heterogeneity of the available literature, variability in study designs and patient cohorts, and the need to integrate findings across diverse clinical settings. This format allows for a more flexible and clinically meaningful synthesis of emerging treatment strategies in advanced prostate cancer.

Androgen Deprivation Therapy (ADT)

Table 1 provides a summary of key studies that assess ADT and its combinations, offering insights into clinical outcomes, quality of life considerations, and economic value in various patient populations.

**Table 1:** Studies Evaluating ADT and Emerging Modalities in Prostate Cancer This table summarizes pivotal studies investigating ADT strategies in prostate cancer, including monotherapy and combination regimens with agents such as enzalutamide. It highlights study design, sample sizes, treatments administered, follow-up durations, and principal outcomes, ranging from biochemical recurrence control and cost-effectiveness to quality-of-life and mental health implications. N/A indicates fields where certain study parameters, such as sample size or follow-up, were not applicable due to the nature of the study ADT: androgen deprivation therapy, OS: overall survival, N/A: not applicable, HSPC: hormone-sensitive prostate cancer, nmCSPC: non-metastatic castration-sensitive prostate cancer, QALYs: quality-adjusted life years, QoL: quality of life, HR: hazard ratio

Study	Study design	Sample size	Treatment	Median follow-up (year)	Study outcomes
Shelan et al. (2024) [7]	Systematic review of prospective trials	N/A	Enzalutamide in non-metastatic HSPC	Varied (0.5-4 years)	Enzalutamide significantly delays biochemical recurrence; favorable safety profile in non-metastatic hormone-sensitive settings.
Aprikian et al. (2025) [6]	Cost-effectiveness study	N/A	Enzalutamide + ADT versus ADT alone	104.45 months	Enzalutamide + ADT is cost-effective for high-risk biochemically recurrent nmCSPC; improved QALYs.
Alwhaibi et al. (2022) [8]	Literature review	N/A	ADT alone	N/A	ADT is associated with increased risk of depression; the importance of pharmacological and psychological management strategies is highlighted.
Gagliano-Jucá et al. (2018) [9]	Prospective cohort	71	ADT monotherapy	1 year	ADT increased pain sensitivity and worsened mood scores; negative impact on QoL underscores the need for supportive care alongside ADT.
Fizazi et al. (2019) [15]	Phase 3, multicenter, randomized, double-blind	1,199	Abiraterone + prednisone + ADT versus placebo + ADT	4.3 (51.8 months)	The abiraterone group had significantly longer OS (53.3 versus 36.5 months; HR: 0.66; p<0.0001); manageable toxicity profile.
Kyriakopoulos et al. (2018) [16]	Phase 3, multicenter, randomized	790	Docetaxel + ADT versus ADT alone	4.5 (53.7 months)	OS benefit in high-volume disease (51.2 versus 34.4 months; HR: 0.63; p<0.001); no OS benefit in low-volume disease (HR: 1.04).
Parker et al. (2018) [17]	Phase 3, multicenter, open-label randomized	2,061	Radiotherapy + ADT ± docetaxel versus ADT ± docetaxel	~3.8 (median not explicitly stated)	Radiotherapy improved failure-free survival (HR: 0.76; p<0.0001), but not OS in the overall population (HR: 0.92; p=0.266); well tolerated.

Study populations included a mix of non-metastatic and biochemically recurrent prostate cancer patients, with high-risk features such as elevated prostate-specific antigen (PSA) levels (≥20 ng/mL), Gleason score ≥ 8, and/or T3-T4 staging. Per STAMPEDE and CHAARTED criteria, metastatic hormone-sensitive prostate cancer was stratified into low-volume disease (oligometastatic: ≤3 bone lesions and no visceral metastases) and high-volume disease (≥4 bone lesions with at least one beyond the axial skeleton or any visceral metastases). High‑volume cases were further subclassified as hormone‑sensitive (HSPC) when responsive to initial ADT or castration‑resistant (CRPC) when progression occurred despite castrate testosterone levels. Interventions ranged from ADT monotherapy to combination therapies involving novel hormonal agents such as enzalutamide, abiraterone, and supportive care considerations.

According to the base-case outcomes in the study by Aprikian et al., the OS for the enzalutamide combination arm also continued to be significantly longer than the ADT alone arm [6]. The estimated mean and median survival in combination with enzalutamide were 160.85 and 148.51 months, respectively, compared to 115.22 and 104.45 months in the selected ADT only. The mean and median survival differences of the two arms were 45.63 and 44.06 months, respectively.

In the same primary treatment setting, the phase 2 EMBARK trial (reported in Shelan et al. [7]) explored the combination of androgen deprivation therapy (ADT) with enzalutamide prior to consideration of radical prostatectomy (RP) [6]. However, this approach remains experimental and is unlikely to result in a paradigm shift in current clinical practice. In the salvage setting, the addition of enzalutamide to standard-of-care (SOC) therapy necessitates a careful evaluation of its clinical utility and justification for early consent. Specifically, this strategy warrants further investigation through comparative studies assessing outcomes among patients treated with RP at low prostate-specific antigen (PSA) levels who subsequently experience biochemical relapse.

In another study of a similar primary setting, there is an increasing interest in combining ADT with enzalutamide pre-RP. The toxicity could not be reduced, although the efficacy of enzalutamide appears to be at least as good as ADT in the EMBARK trial [7]. It is so tempting to assume that since enzalutamide does not reduce testosterone levels, it is more tolerable than ADT. Grade 3 or greater adverse events were also found in 50% of patients receiving enzalutamide monotherapy in the EMBARK trial compared to 42.7% in leuprolide. By looking into the type of adverse events based on the arm of treatment, it is clear that the hot flashes in the leuprolide arm were more common than in the enzalutamide arm (57.3% versus 21.8%). Quite the opposite, fatigue was more present in the enzalutamide arm than in the leuprolide arm (46.6% versus 32.8%), and so were gynecomastia (44.9% versus 9.0%) and ischemic heart disease (9.0% versus 5.6%).

Overall and on the basis of the data on the incidence of depression offered by Alwhaibi et al. [8], the average incidence of depression is 18.23% (range: 2.1%-46.9%) in prostate cancer (PCa) patients who receive ADT compared to 8.42% (range: 1.4%-23.3%) in the non-ADT patients. Collectively, these findings support the hypothesis that ADT is associated with an elevated risk of depression in PCa patients, which may, in turn, negatively impact treatment adherence and overall disease management.

In the study conducted by Gagliano-Jucá et al., a total of 77 participants were enrolled, including 37 individuals in the ADT group [9]. The cohort had an average body mass index (BMI) of 28.3 kg/m^2^ (range: 21.9-37.7 kg/m^2^), and the median age was 67 years (range: 53-89 years). At baseline, serum total testosterone levels were within the normal range in both groups. As anticipated, participants scheduled to receive ADT exhibited higher Gleason scores compared to those in the non-ADT group, and a greater proportion of men in the ADT group also underwent radiation therapy.

The three pivotal phase 3 trials evaluated intensified treatment strategies in mHSPC [15-17]. LATITUDE demonstrated significantly prolonged overall survival with abiraterone plus prednisone and ADT in high-risk patients. CHAARTED showed a survival benefit of adding docetaxel to ADT, especially in those with high-volume disease, while low-volume patients saw no OS improvement. STAMPEDE revealed that prostate radiotherapy improved failure-free survival, but not overall survival in the unselected mHSPC population. Notably, radiotherapy was well tolerated. Together, these studies support tailoring therapy based on disease burden and risk, with systemic intensification offering clear survival gains in high-volume or high-risk cases.

Chemotherapy (Docetaxel and Cabazitaxel)

Table 2 summarizes key studies exploring the clinical efficacy, patient-reported outcomes, and contextual considerations in the use of these agents for mCRPC.

**Table 2:** Studies on Cabazitaxel and Docetaxel Use in mCRPC This table compiles selected studies evaluating the use of docetaxel and cabazitaxel in patients with mCRPC. It includes randomized trials, retrospective analyses, and narrative reviews. Data presented cover study design, sample size (where applicable), treatment regimens, median follow-up durations, and primary outcomes, including OS, PFS, treatment tolerability, and patient preference. “N/A” indicates that certain parameters were not applicable based on the study type, not due to missing data. mCRPC: metastatic castration-resistant prostate cancer, OS: overall survival, PFS: progression-free survival, N/A: not applicable, ARTA: androgen receptor-targeted agent, PFS: progression-free survival, ARAT: androgen receptor axis-targeting

Study	Study design	Sample size	Treatment	Median follow-up (year)	Study outcomes
Oudard et al. (2017) [10]	Randomized phase 3 trial	1,168	Cabazitaxel versus docetaxel (first-line mCRPC)	2.6	No OS difference; similar efficacy; better tolerability with cabazitaxel.
Baciarello et al. (2022) [18]	Randomized trial (patient preference study)	173	Patient preference: cabazitaxel versus docetaxel	1.5	Patients preferred cabazitaxel due to perceived tolerability.
Suzuki et al. (2025) [19]	Narrative review	N/A	Review of cabazitaxel evidence in prostate cancer	N/A	Summarizes the effectiveness of cabazitaxel post-ARTA failure.
Shiota et al. (2020) [20]	Retrospective study	47	Cabazitaxel post-docetaxel with/without prior ARAT	N/A	Prior ARAT use is associated with shorter PFS in cabazitaxel-treated patients.

According to Oudard et al., the incidence of grade 3 or 4 treatment-emergent adverse events (TEAEs) was reported as 41.2% in the C20 group, 60.1% in the C25 group, and 46% in the D75 group [10]. Patients receiving C25 experienced higher rates of febrile neutropenia, neutropenic infections, diarrhea, and hematuria, whereas those in the D75 group had increased occurrences of peripheral neuropathy, stomatitis, peripheral edema, alopecia, and nail disorders. Discontinuation of treatment due to TEAEs occurred in 25.2% of patients in the C20 group, 31.7% in the C25 group, and 33.9% in the D75 group. Within 30 days of the final treatment dose, mortality rates were 2.7% (12 patients) for C20, 4.1% (16 patients) for C25, and 3.1% (10 patients) for D75, with some deaths attributable to adverse events. Specifically, three patients (0.8%) in the C20 group, 11 (2.8%) in C25, and eight (2.1%) in D75 died due to treatment-related AEs. Adverse events contributing to more than one death across treatment arms included myocardial infarction, sepsis, and neutropenic sepsis. Prophylactic use of granulocyte colony-stimulating factor (G-CSF) was more frequent in the C25 group (1,205 cycles) compared to C20 (564 cycles) and D75 (667 cycles). In treatment cycles where prophylactic G-CSF was administered, the incidences of all-grade, grade 3, and grade 4 neutropenia were 6%, 1.2%, and 0.4% for C20; 10.9%, 3.7%, and 3.2% for C25; and 8.7%, 2.2%, and 3.1% for D75, respectively.

Baciarello et al. noted that investigators were not involved in the selection or administration of study medications, which eliminated bias in drug preference and reduced the necessity for blinding [18]. Furthermore, the differential requirement for high-dose steroid premedication with docetaxel would have likely revealed treatment allocation, making effective blinding impractical without the use of placebo controls. A key limitation of the study design was that cabazitaxel was only compared to docetaxel at the 25 mg/m^2^ dose (C25), as this is the standard reference dose in Europe. However, findings from the PROSELICA trial demonstrated that a reduced dose of cabazitaxel (C20) was non-inferior to C25 in terms of efficacy, suggesting that C20 may offer improved tolerability and potentially influence the perceived differences in safety profiles between the taxanes. Additionally, a higher proportion of patients in the cabazitaxel-first arm did not proceed to receive the second taxane (20.4% versus 8.4% in the docetaxel-first arm), leading to a total dropout rate of 22.1% after the first treatment period. Nonetheless, the trial design anticipated that disease progression would occur during the initial treatment phase, and the resulting attrition was accounted for in the statistical power calculations, preserving the validity of the study outcomes.

Suzuki et al. demonstrated that the efficacy of cabazitaxel in older patients with metastatic castration-resistant prostate cancer (mCRPC) who had progressed following docetaxel therapy was consistent with findings from international studies and similarly validated in Japan [19]. Post-marketing surveillance revealed that patients aged 80 years and older received cabazitaxel and generally had treatment histories comparable to those of younger patients. Although real-world data indicate that older patients in Japan are less likely to receive the full initial dose of cabazitaxel, the dose administered per cycle did not differ significantly between age groups. The incidence of adverse drug reactions (ADRs) was comparable, with 77.2% reported in patients under 80 and 79.6% in those aged 80 and above. Likewise, the rate of febrile neutropenia showed no significant elevation in the older cohort (<80 years: 48.4%; ≥80 years: 57.1%). These findings suggest that clinicians in Japan likely individualize cabazitaxel dosing based on patient-specific clinical conditions. Additionally, baseline characteristics associated with early treatment discontinuation, such as the presence of liver metastases, poor Eastern Cooperative Oncology Group performance status (ECOG PS), and elevated prostate-specific antigen (PSA) levels, were observed in the post-marketing population of Japanese mCRPC patients.

Shiota et al. conducted a study on cabazitaxel chemotherapy clinical outcome on patients with or without exposure to androgen receptor axis-targeting (ARAT) agents [20]. PSA response, progression-free survival, and overall survival of mCRPC patients who were treated with cabazitaxel-based chemotherapy were similar between those with prior use of ARAT and those without. Cabazitaxel is proposed to be effective as it retains the antimicrobial activity following the application of ARAT agents as opposed to docetaxel. Nevertheless, the following aspects should be considered: (i) cabazitaxel is conventionally used in the post-docetaxel setting, (ii) the general dose of the cabazitaxel agent (25 mg/m^2^) is not equal to that of the docetaxel agent (75 mg/m^2^), and (iii) although ARAT agents were clinically available in Japan when the cabazitaxel agent was accepted, docetaxel agent was already accepted in advance.

The PEACE‑1 trial enrolled 1,173 men with de novo high-volume metastatic hormone‑sensitive prostate cancer and randomized them to ADT + docetaxel with or without abiraterone [21]. At a median follow-up of 3.5 years, the addition of abiraterone to ADT + docetaxel significantly improved radiographic progression-free survival and overall survival, establishing triplet therapy as a new standard for fit patients with high-volume disease.

Novel Hormonal Agents (Abiraterone and Enzalutamide)

Table 3 provides an overview of recent studies comparing abiraterone and enzalutamide in mCRPC, with a focus on cardiovascular and metabolic risks, healthcare utilization, and survival outcomes.

**Table 3:** Studies Evaluating Abiraterone and Enzalutamide in mCRPC Patients This table summarizes key retrospective studies assessing abiraterone and enzalutamide in the mCRPC setting. Study parameters include data source, sample size, treatment arms, follow-up duration, and primary outcomes. The findings highlight comparative risks (e.g., cardiovascular/metabolic events), healthcare costs, and OS. “N/A” is not used in this table, as all relevant data points are applicable and reported. mCRPC: metastatic castration-resistant prostate cancer, OS: overall survival, N/A: not applicable, HR: hazard ratio, CI: confidence interval

Study	Study design	Sample size	Treatment	Median follow-up (year)	Study outcomes
Lai et al. (2022) [21]	Retrospective cohort (SEER-Medicare database)	6,387	Abiraterone or enzalutamide	~2.5	Higher cardiovascular/metabolic event risk with abiraterone versus enzalutamide (HR: 1.34; 95% CI: 1.14-1.56).
Ramaswamy et al. (2020) [22]	Retrospective claims-based analysis	3,204	Enzalutamide versus abiraterone + prednisone	~1.5	Enzalutamide had lower overall healthcare costs and reduced hospitalization risk versus abiraterone.
Jarimba et al. (2021) [23]	Retrospective single-center study	80	Enzalutamide versus abiraterone	1.2	Median OS: 18.2 months (enzalutamide) versus 16.4 months (abiraterone); not statistically significant.

Lai et al. analyzed a cohort of 56,230 men with advanced prostate cancer to assess the primary composite outcomes associated with abiraterone and enzalutamide therapies [21]. Of this population, 2,736 patients received abiraterone and 2,466 received enzalutamide. The baseline characteristics of patients who had ever received either therapy were comparable in terms of age, race, socioeconomic status, and comorbidity burden. Notably, those treated with abiraterone or enzalutamide were slightly younger, with median ages of 76.8 and 78.8 years, respectively, and demonstrated better overall health status, with 49.6% and 44% having a comorbidity score of zero, compared to those who had never received these agents. These findings offer valuable insights into patient selection and real-world usage patterns of novel hormonal agents in the management of advanced prostate cancer, especially as clinical strategies involving abiraterone and enzalutamide continue to evolve.

Ramaswamy et al. evaluated healthcare utilization and cost outcomes among 1,229 men with metastatic castration-resistant prostate cancer (mCRPC) treated with enzalutamide and 1,945 treated with abiraterone, with mean ages of 74 and 73 years, respectively [22]. After propensity score matching (PSM), each treatment cohort included 1,160 patients. Enzalutamide use was associated with significantly fewer all-cause outpatient visits (2.51 versus 2.86; p<0.0001) and prostate cancer (PCa)-related outpatient visits (0.86 versus 1.03; p<0.0001) compared to abiraterone. Additionally, all-cause outpatient costs ($2,588 versus $3,115; p<0.0001) and PCa-related outpatient costs ($1,356 versus $1,775; p<0.0001) were significantly lower in the enzalutamide group. The total monthly costs per patient (PPPM), including medical and pharmacy expenses, were also lower with enzalutamide for both all-cause ($8,085 versus $9,092; p=0.0002) and PCa-related spending ($6,321 versus $7,280; p<0.0001). Notably, corticosteroid use in the pre-index period was more prevalent in patients treated with abiraterone (45.69%) than in those treated with enzalutamide (26.90%).

In a separate analysis, Jarimba et al. reported a significantly higher biochemical response rate with enzalutamide compared to abiraterone (77.1% versus 58.1%; p=0.016) among patients with mCRPC [23]. Furthermore, achieving a biochemical response was associated with reduced risks of biochemical progression (odds ratio (OR): 0.248; p=0.017) and mortality (OR: 0.302; p=0.038). Although other clinical outcomes appeared to favor enzalutamide, the differences did not reach statistical significance. These findings underscore the potential clinical and economic advantages of enzalutamide over abiraterone and highlight the need for prospective trials to further elucidate their comparative efficacy.

Radiotherapy (Targeted RT)

Table 4 highlights key clinical and review-based studies evaluating the role of radiotherapy in stage IV prostate cancer, focusing on therapeutic outcomes, toxicity profiles, and biological correlates.

**Table 4:** Studies Evaluating Radiotherapy in Stage IV Prostate Cancer This table presents selected studies exploring the application of radiotherapy in advanced prostate cancer. It includes prospective clinical studies and narrative reviews addressing RT either alone or in combination with systemic treatments. Information includes study design, sample size (if applicable), type of radiotherapy intervention, follow-up duration, and primary outcomes, such as treatment toxicity, immunomodulatory effects, and predictive biomarkers such as CTCs. “N/A” denotes elements not applicable to the study type. RT: radiotherapy, CTC: circulating tumor cell, CETC: circulating epithelial tumor cells, N/A: not applicable

Study	Study design	Sample size	Treatment	Median follow-up (year)	Study outcomes
Konnerth et al. (2024) [24]	Prospective cohort	213	Targeted therapies + RT	1.2	Low early toxicity; most adverse events were manageable and grade 1-2 only.
Schaue and McBride (2015) [25]	Narrative review	N/A	RT in various cancers	N/A	RT was effective when combined with systemic therapy; immune response modulation.
Schott et al. (2025) [26]	Prospective study	98	RT + CTC monitoring	2	CETC count correlated with recurrence risk; high CETC linked to early relapse.

Konnerth et al. reported that patients received a total of 683 radiation therapy (RT) cycles in combination with 51 types of targeted therapy (TT) agents [24]. Among these RT series, 314 (46%) were administered without stopping targeted therapy or with less than a one-week interruption prior to irradiation. Additionally, in 106 RT series (16%), chemotherapy was concurrently administered with targeted therapy. RT treatments were applied to various anatomical regions, including the head and neck (6%), central nervous system (25%), thoracic region (32%), breast (8%), abdominal/pelvic region (24%), and extremities (4%).

Schaue and McBride found that radiotherapy alone is unlikely to reverse the negative immune microenvironment present in most tumors and may, in fact, reinforce immunosuppressive conditions [25]. Radiation has been observed to increase TREG cell representation and promote the infiltration of M2 macrophages. In certain preclinical models, radiation-induced vascular damage, possibly enhanced by stereotactic body radiation therapy (SBRT) or charged particle therapy (CPT), has been associated with chronic hypoxia. This environment facilitates the influx of CD11b+ myeloid cells that develop into M2 growth-promoting macrophages in chronically hypoxic areas. Radiation-induced myeloid cell infiltration can be inhibited by targeting the hypoxia-inducible factor-1 alpha/stromal cell-derived factor-1/C-X-C chemokine receptor type 4 (HIF-1α/SDF-1/CXCR4) or colony-stimulating factor 1/colony-stimulating factor 1 receptor (CSF1/CSF1R) pathways, leading to tumor radiosensitization. These approaches are currently being evaluated in clinical trials. Additionally, such treatments impede angiogenesis, increasing reliance on less efficient vasculogenesis in irradiated tissues and tumors.

Schott et al. examined the relationship between changes in circulating epithelial tumor cells (CETCs/CTCs) and the risk of relapse in prostate cancer patients [26]. CETCs/CTCs were identified in 96% of patients prior to radiotherapy (RT). The study found that a decrease in CETC/CTC counts during RT, particularly in surgically treated patients, was associated with a reduced risk of relapse. In contrast, an increase in CETC/CTC counts during or after RT was linked to a significantly higher risk of relapse (hazard ratio (HR): 8.8; p=0.002). Among patients receiving adjuvant RT, 36% relapsed, compared to 14% in those undergoing definitive RT, with higher relapse rates observed in patients whose CETC/CTC counts increased during treatment. In the salvage RT group, 18% experienced relapse; however, changes in CETC/CTC counts did not show a strong correlation with relapse in this cohort. The study suggests that monitoring CETC/CTC levels during RT may offer valuable prognostic insight into relapse risk in prostate cancer patients.

Discussion

Comparative analyses of treatment strategies for stage IV prostate cancer indicate that no single therapeutic approach universally applies to all patients; instead, treatment efficacy depends on individual patient characteristics, disease burden, and comorbidities. ADT remains foundational across various stages of the disease. The EMBARK trial and systematic reviews show that combining ADT with agents such as enzalutamide improves biochemical control, especially in high‑risk non‑metastatic cases. Early intensification with novel hormonal agents is especially beneficial for patients with biochemical recurrence or nmCSPC, although clinicians must consider the associated side effects, including fatigue and cardiovascular risks, to ensure personalized treatment.

Landmark clinical trials, such as STAMPEDE, CHAARTED, LATITUDE, and ENZAMET, have reshaped first-line management of hormone-sensitive metastatic prostate cancer, particularly for high-volume or de novo metastatic disease, by demonstrating the survival benefits of early intervention with docetaxel or abiraterone [15-17,27]. Nonetheless, patient preference and treatment tolerability remain essential considerations, as illustrated in the CABADOC study, which found that patients favored cabazitaxel over docetaxel due to perceived tolerability. Economic considerations also influence treatment decisions. Ramaswamy et al. reported lower healthcare costs associated with enzalutamide compared to abiraterone [22], while Aprikian et al. demonstrated the cost-effectiveness of enzalutamide combined with ADT in the Canadian healthcare context [6].

Maintaining quality of life is especially vital in older patients and those with multiple comorbidities. ADT has been associated with increased risk of depression and decreased vitality, highlighting the importance of supportive care. Therapies should align with performance status and comorbidities: chemotherapy for fit patients with aggressive disease and hormonal therapy for frail patients or those with cardiovascular risks. Additionally, high-resolution radiotherapy, supported by biomarkers such as circulating epithelial tumor cells (CETCs) or circulating tumor cells (CTCs), may offer promising adjuvant benefits in selected patients.

Abiraterone acts as a selective and irreversible inhibitor of CYP17A1, a critical enzyme in the steroidogenesis pathway responsible for androgen production in the testes, adrenal glands, and tumor microenvironment [28]. By inhibiting CYP17A1, abiraterone reduces circulating levels of testosterone and dihydrotestosterone (DHT), thereby decreasing the availability of ligands for androgen receptors (AR) [28]. To mitigate mineralocorticoid-related adverse effects, abiraterone is co-administered with low-dose corticosteroids [29]. Its use is most beneficial in patients without androgen receptor splice variant 7 (AR-V7) expression and those who have not been heavily pretreated with other AR pathway inhibitors [29].

Enzalutamide, on the other hand, is a potent AR signaling inhibitor that directly binds to the ligand-binding domain (LBD) of the AR [30]. It impedes several steps in AR signaling by blocking androgen binding, preventing nuclear translocation, inhibiting DNA binding, and precluding coactivator recruitment, thereby suppressing AR-mediated transcription [30]. However, resistance to enzalutamide is commonly associated with the presence of the AR-V7 splice variant, which lacks the LBD, enabling the receptor to remain constitutively active in the nucleus despite the absence of ligand or AR antagonists [30].

Docetaxel, a taxane-based chemotherapeutic agent, exerts its antitumor effects by stabilizing microtubules through the promotion of tubulin polymerization and the inhibition of depolymerization, resulting in mitotic arrest and apoptosis [31]. Importantly, docetaxel has also been shown to interfere with AR signaling by preventing AR nuclear translocation, a property that may be advantageous in tumors harboring certain AR mutations or splice variants [31].

Clinical decision-making regarding first-line therapy in advanced prostate cancer should consider the underlying AR biology, including AR-V7 expression and genomic alterations. For AR-V7-negative castration-resistant prostate cancer (CRPC), enzalutamide or abiraterone remains a viable option [30]. In contrast, patients with AR-V7-positive disease may benefit more from docetaxel or other taxane-based treatments. Furthermore, individuals with DNA repair deficiencies may derive greater therapeutic benefit from docetaxel, given its broader mechanism of action that bypasses AR dependency [31].

Clinical Guideline Snapshot

Contemporary clinical guidelines issued by the National Comprehensive Cancer Network (NCCN), European Association of Urology (EAU), and American Urological Association (AUA) consistently recommend androgen deprivation therapy (ADT) as the first-line treatment for stage IV prostate cancer, with escalation to additional agents such as docetaxel or novel hormonal therapies such as abiraterone or enzalutamide based on disease progression and metastatic burden. While these recommendations maintain brevity in therapeutic sequencing, they uniformly emphasize the importance of shared decision-making, considering patient-specific factors such as performance status, comorbidities, and treatment preferences. In this context, abiraterone, a selective and irreversible CYP17A1 inhibitor that suppresses androgen biosynthesis in the testes, adrenal glands, and tumor microenvironment, is recognized for its ability to reduce circulating testosterone and dihydrotestosterone levels, thereby limiting ligand availability to the androgen receptor (AR) [28]. To mitigate mineralocorticoid-related adverse events, abiraterone is co-administered with low-dose corticosteroids [29]. Its clinical utility is especially pronounced in patients who are AR-V7-negative and have not undergone extensive prior exposure to AR-targeted therapies, thereby optimizing its therapeutic efficacy.

Limitations

This narrative review delivers contextual depth and interpretive flexibility but lacks the methodological rigor and reproducibility of systematic reviews, introducing potential selection bias. The paucity of head‑to‑head randomized trials comparing key modalities (e.g., enzalutamide versus abiraterone) limits our ability to name a superior option definitively. Furthermore, variability in clinical practice patterns, physician expertise, and patient demographics constrains the generalizability of trial results to everyday settings. Future research should prioritize large‑scale, comparative trials and robust real‑world evidence studies to refine and personalize treatment recommendations.

## Conclusions

The management of stage IV prostate cancer requires a personalized, patient-centered approach that accounts for disease burden, comorbidities, treatment tolerability, and individual preferences. Evidence increasingly supports the use of combination therapies, such as androgen deprivation therapy (ADT) with novel hormonal agents or chemotherapy regimens, as they offer improved clinical outcomes in appropriately selected patients. As therapeutic paradigms continue to evolve with ongoing research and emerging evidence, there is growing potential to enhance survivorship and quality of life through more targeted, individualized treatment strategies.
